# Brain-penetrant calcium channel blockers are associated with a reduced incidence of neuropsychiatric disorders

**DOI:** 10.1038/s41380-022-01615-6

**Published:** 2022-05-26

**Authors:** Lucy Colbourne, Paul J. Harrison

**Affiliations:** 1 Department of Psychiatry, University of Oxford, Oxford, OX3 7JX UK; 2 Oxford Health NHS Foundation Trust, Warneford Hospital, Oxford, OX3 7JX UK

**Keywords:** Depression, Predictive markers

## Abstract

Calcium channel blockers (CCBs) differ in their ability to penetrate into the brain. Pharmacoepidemiological studies suggest that CCBs as a class may have beneficial effects on the risks and outcomes of some psychiatric and neurological disorders. It is plausible but unknown whether this effect relates to their brain penetrance. To address this, we used the TriNetX electronic health records network to identify people prescribed a brain-penetrant CCB (BP-CCB), or those given amlodipine, a CCB with low brain penetrability. We created cohorts of patients who, prior to first CCB exposure, either had to have, or could not have had, a recorded ICD-10 diagnosis in any of the following categories: psychotic disorder; affective disorder (including bipolar disorder and major depressive disorder); anxiety disorder; substance use disorder; sleep disorder; delirium; dementia, or movement disorder. Cohort pairs were propensity score matched for age, sex, race, blood pressure, body mass index, and a range of other variables. The outcomes were the incidence of these disorders measured over a two-year exposure period. Matched cohort sizes ranged from 17,896 to 49,987. In people with no prior history of psychiatric or neurodegenerative disorder, there was a significantly lower incidence of most disorders with BP-CCBs compared to amlodipine, with risk ratios ranging from 0.64 to 0.88 and an overall risk ratio of 0.88, i.e. a risk reduction of 12%. In people who did have a prior psychiatric or neurodegenerative diagnosis, differences were much smaller, but again showed lower risks for several disorders with BP-CCBs compared to amlodipine. The differences were somewhat more marked in women and in people less than 60 years old. Results were similar when comparing BP-CCBs with verapamil and diltiazem. We also compared BP-CCBs with angiotensin receptor blockers, and found an overall risk ratio of 0.94 in favour of BP-CCBs, but with differential effects across disorders including a higher risk of psychotic disorder and dementia, but a lower risk for anxiety and sleep disorders. In some analyses, there was evidence of residual confounding even after the extensive matching, in that negative control outcomes showed a reduced incidence with BP-CCBs relative to the comparator cohort. In summary, CCBs that readily penetrate the brain are associated with a lower incidence of neuropsychiatric disorders, especially first diagnoses, compared to CCBs which do not. This may reflect their blockade of neuronal voltage-gated calcium channels. The findings encourage repurposing trials using existing BP-CCBs, and suggest that novel BP-CCBs with enhanced and more selective central actions might have greater therapeutic potential for psychiatric and neurodegenerative disorders.

## Introduction

The main antihypertensive drug classes are calcium channel blockers (CCBs), diuretics, angiotensin converting enzyme inhibitors (ACEIs), angiotensin II receptor blockers (ARBs), and β-blockers. CCBs target the α1 subunits of L-type voltage-gated calcium channels, especially Ca_V_1.2 and Ca_V_1.3, encoded by *CACNA1C* and *CACNA1D* respectively [1, 2]. There has been debate as to whether antihypertensive drugs impact the onset or course of neuropsychiatric disorders. This particularly applies to CCBs, because of the hypothesized role of calcium signalling in their pathophysiology [3, 4], and by the results of some early clinical studies [5–7]. Although no good evidence of psychotropic efficacy materialised [8, 9], interest in the possibility has been rekindled by the discovery that voltage-gated calcium channel subunits are genome-wide significant risk genes for schizophrenia and bipolar disorder [2, 10].

One contemporary approach has been to use electronic health records to examine whether CCBs are associated with differential incidences or outcomes of neuropsychiatric disorders. For example, in the Swedish population, patients with serious mental illnesses had lower rates of psychiatric hospitalization and self-harm when they were taking CCBs than when they were not [11]. Other studies have compared CCBs with one or more of the other antihypertensive drug classes. Results are variable, but the literature overall suggests that, for major psychiatric disorders, CCBs are associated with lower incidences than β-blockers, are broadly comparable to diuretics and ACEIs, but have a higher incidence than with ARBs [12–18]. A similar approximate ranking applies to delirium [19] and to neurodegenerative and movement disorders [20–23], but see also ref. [24].

Notably, individual CCBs differ in their ability to cross the blood brain barrier. As introduced below, most do enter the brain, but amlodipine, much the most widely used drug in the class, does not do so to any significant extent. Whilst any effects of CCBs on neuropsychiatric disorders could be mediated peripherally, it is more plausible that such effects would result from occupancy of neuronal voltage-gated calcium channels in the brain [25–27]. Few studies to date have investigated whether brain-penetrant CCBs (BP-CCBs) have greater effects on brain disorders than those which are non-penetrant, although two studies reported a decreased risk of Parkinson’s disease with BP-CCBs compared to amlodipine [28, 29].

Here, we used an electronic health records network to investigate whether BP-CCBs are associated with reduced incidence of a first diagnosis, or a subsequent diagnosis, of common psychiatric and neurodegenerative disorders compared to amlodipine over a two-year exposure period. Extensive propensity score matching was used to reduce confounding. We conducted secondary analyses to assess the robustness of the results and used negative control outcomes to aid their interpretation.

## Materials and methods

### Brain penetrability of CCBs

CCBs are recognised to differ in their blood-brain barrier permeability and thence their potential occupancy of brain voltage-gated calcium channels. CCBs also differ structurally; most are dihydropyridines, except for the two earliest CCB drugs, verapamil (a phenylalkylamine) and diltiazem (a benzothiazepine). For the primary analysis, the comparison was limited to dihydropyridines, as explained below. Our primary question was to ask whether brain penetrability impacts on the neuropsychiatric correlates of CCB use. We acknowledge that there is a spectrum of brain-penetrability, and also that evidence is often incomplete [30, 31]. However, for the purposes of this study, we dichotomised the dihydropyridine CCBs into those generally considered to have high brain penetrability and those which do not. For simplicity, we describe these groups as ‘brain-penetrant’ (BP-CCB) and ‘non-penetrant’ respectively. This distinction resulted in amlodipine being assigned as non-penetrant, and the other dihydropyridine CCBs (felodipine, isradipine, nicardipine, nifedipine, nimodipine, nisoldipine) being assigned as brain-penetrant, in line with prior categorisations, [28, 29, 32–34] which were in turn based on a range of experimental data (see Supplementary Table 1) [34–47].

### TriNetX electronic health records network

TriNetX Analytics is a cloud-based federated electronic health records network with over 85 million patients in a range of healthcare organizations, mostly in the USA. Full details about the network and its data can be found in ref. [48]. Briefly, via a browser interface, the user can view aggregated and de-identified data, such as demographics, diagnostics (ICD-10 codes), medications, and lab values, and create cohorts based on combinations of inclusion and exclusion variables, match and compare pairs of cohorts, and conduct statistical analyses to explore cohorts and differences between them. The process by which the data in the TriNetX network are de-identified is attested to through a formal determination by a qualified expert as defined in Section #164.514(b)(1) of the HIPAA Privacy Rule [48].

### Cohorts and covariates

The basic design of cohorts and selection of covariates was as described [17]. We created two types of cohort, both open to all patients aged between 18 and 90 years old. In the first, we excluded patients who had any prior psychiatric or neurodegenerative diagnosis (see below for list of ICD-10 codes), in order to assess the effect of BP-CCB versus amlodipine on a first psychiatric or neurodegenerative diagnosis. In the second type of cohort, we only included patients who *did* have a diagnosis of this kind prior to exposure, in order to investigate the effects on recurrence. Note that for the latter cohort, the diagnosis during the exposure period did not have to be the same diagnosis as that prior to exposure (e.g. a patient could be included in the cohort due to an existing diagnosis of anxiety disorder and be diagnosed with a psychotic disorder during the exposure period).

Without matching, BP-CCB and amlodipine cohorts differed significantly in age, sex, blood pressure and prior exposure to other antihypertensives (data not shown) and we used propensity score matching to minimise these and other potential confounding factors before conducting analyses [49, 50]. Thus, all cohorts were matched at baseline (i.e. before their first exposure to the drugs of interest) for age, sex, race, blood pressure, body mass index, and prior prescription of: non-CCB antihypertensives, antidepressants, antipsychotics, anxiolytics, gabapentinoids, lithium, stimulants and levodopa. In addition, they were matched for a history of hypertensive disease (ICD-10 code I10-I16), ischaemic heart disease (I20-I25), thyroid disease (E00-E07), diabetes mellitus (E08-E13), disorders of the respiratory system (J00-J99), disorders of the musculoskeletal system (M00-M99), and problems related to socioeconomic and psychosocial circumstances (Z55-Z65). Matching was carried out within TriNetX using 1:1 greedy nearest neighbour propensity score matching [48]; a standard difference of 0.1 between cohorts for a variable is considered negligible [51].

### Outcomes

The outcomes of interest were diagnoses of the major adult psychiatric disorders and common neurodegenerative disorders: psychotic disorders (F20-F29), affective disorders (F30-F39), anxiety disorders (F40–48), substance use disorder (F10-F19), sleep disorder (F51, G47), delirium (F05, R40.0, R41.0), dementia (F01-F03, G30, G31.0, G31.2, G31.83) and movement disorder (G20-G26). We also measured schizophrenia (F20), bipolar disorder (F31), and major depressive disorder (F32, F33) separately. We investigated twelve negative control outcomes (i.e. outcomes for which there are no known or predicted links to the brain penetrability of CCBs) to help assess residual confounding [52, 53].

The exposure period of interest was two years. Exposure during this time was proxied by requiring prescriptions at least two years apart for a BP-CCB, or amlodipine; the individual BP-CCB could vary during this period. We did not restrict prescribing of other drugs (e.g. additional antihypertensives), in order to enhance the real-world generalisability of the findings.

We measured the incidence (percentage) of patients receiving a diagnosis during the two year period, and compared matched cohorts using the risk ratio (RR) with 95% confidence intervals. Statistical analyses were conducted within TriNetX.

### Secondary analyses

Differences found between BP-CCBs and amlodipine might reflect something unique about amlodipine rather than merely its low brain penetrability, such as aspects of its channel blocking kinetics [54] Thus, in a secondary analysis we compared people prescribed BP-CCBs with patients prescribed verapamil or diltiazem. We omitted these drugs from the primary analysis since (a) they have different clinical indications from amlodipine (i.e. they are not recommended first line for hypertension, and are contraindicated in heart failure) and hence are more likely to lead to confounding by indication, and (b) their pharmacological profiles differ from the dihydropyridines [1, 27]. Though the evidence regarding the brain penetrability and central effects of verapamil and diltiazem is less clear than for the dihydropyridines, we included both drugs in the non-penetrant group, in line with others [29, 33, 34].

We also conducted secondary analyses for BP-CCBs versus amlodipine separately in men and women, and based on age (under or over 60 years). Finally, we compared BP-CCBs with ARBs, given the evidence mentioned earlier that the latter group are associated with lower risks of many neuropsychiatric disorders compared to CCBs as a class. We hypothesised that this difference would be reduced, or eliminated, when the comparison was limited to BP-CCBs. The same covariates were used for the secondary analyses as for the primary comparison between BP-CCBs and amlodipine.

A STROBE document was completed (Supplementary Table 2).

## Results

Successful matching was achieved for all variables in all cohort pairs, except where noted in a Table. The most commonly prescribed BP-CCB was nifedipine, followed by felodipine and nicardipine; these three drugs together comprised over 97% of BP-CCB prescriptions in each cohort.

### Effects of BP-CCB versus amlodipine on a first neuropsychiatric diagnosis

In this analysis, we excluded all patients who had a prior diagnosis of any of the outcomes of interest. After matching, each cohort had 44,731 patients (Table 1 and Supplementary Table 3A). As shown in panel A of Table 2, in the following two years, those prescribed BP-CCBs had a lower incidence of all diagnoses, although the risk ratio for bipolar disorder included 1, reflecting the low incidence (expected in a population of this age) and thus wide confidence intervals. For a diagnosis of 'any disorder', the relative risk was 12% lower (RR = 0.88 [0.86–0.90]), with an absolute incidence of 21.3% with BP-CCBs and 24.1% with amlodipine. Risk ratios were broadly similar for each individual disorder, ranging from delirium (RR = 0.72 (0.63–0.81) to sleep disorder (RR = 0.88 (0.84–0.91)).

**Table 1:** A: Main baseline demographics of matched cohorts comparing BP-CCBs with amlodipine. A: patients with no prior neuropsychiatric diagnosis. B: patients with a prior neuropsychiatric diagnosis^a^.

	A: no prior neuropsychiatric diagnosis		B: with prior neuropsychiatric diagnosis	
	BP-CCB	Amlodipine	BP-CCB	Amlodipine
Cohort size (*n*)	44,731	44,731	17,896	17,896
Age at index (y, SD)	58.3 (17.5)	58.8 (16.8)	56.2 (17.1)	56.0 (15.7)
Sex (M:F %)	42:58	43:57	38:62	38:62
Race (W,B,O %)^b^	50,29,21	48,28,24	61,26,13	62,26,12
Blood pressure	136/77	136/77	134/77^c^	135/79^c^
Body mass index (SD)	30.1 (6.7)	29.8 (6.7)	30.7 (7.3)	30.4 (7.1)
Prior psychotic disorder (%)	0	0	3	3
Prior affective disorder (%)	0	0	33	33
Prior anxiety disorder (%)	0	0	32	33
Prior substance use disorder (%)	0	0	27	27
Prior sleep disorder (%)	0	0	33	34
Prior delirium (%)	0	0	4	4
Prior dementia (%)	0	0	2	3
Prior movement disorder (%)	0	0	6	6

^a^For additional cohort demographics see Supplementary Table 3. For ICD-10 codes see text. In B, percentages add up to more than 100% since a patient may have a diagnosis in more than one category, and also more than one diagnosis within each category.

^b^W: white. B: black. O: other or not known. SD: standard deviation.

^c^Standard difference between cohorts for diastolic blood pressure = 0.12.

**Table 2:** Outcomes for BP-CCBs versus amlodipine, showing percentage with each diagnosis during the exposure period, and the risk ratio. A: patients with no prior neuropsychiatric diagnosis. B: patients with a prior neuropsychiatric diagnosis.

	A: no prior neuropsychiatric diagnosis			B: with prior neuropsychiatric diagnosis		
	BP-CCB (%)	Amlodipine (%)	Risk ratio (95% CI)	BP-CCB (%)	Amlodipine (%)	Risk ratio (95% CI)
Psychotic disorder	0.4	0.5	**0.76 (0.63–0.93)**	2.5	3.0	**0.83 (0.74–0.94)**
Schizophrenia	0.1	0.2	**0.64 (0.45–0.91)**	1.0	1.3	**0.73 (0.60–0.89)**
Affective disorder	6.3	7.3	**0.86 (0.82–0.90)**	32.6	33.5	0.97 (0.94–1.00)
Bipolar disorder	0.4	0.5	0.82 (0.68–1.00)	3.7	4.2	**0.88 (0.80–0.98)**
Major depressive disorder	5.6	6.6	**0.85 (0.81–0.90)**	29.0	29.7	0.98 (0.95–1.01)
Anxiety disorder	7.1	8.2	**0.86 (0.83–0.90)**	31.0	32.6	**0.95 (0.92–0.98)**
Sleep disorder	8.7	9.9	**0.88 (0.84–0.91)**	31.1	31.6	0.98 (0.95–1.01)
Substance use disorder	5.0	6.2	**0.81 (0.76–0.85)**	23.2	24.7	**0.94 (0.91–0.98)**
Delirium	0.9	1.3	**0.72 (0.63–0.81)**	3.2	3.4	0.95 (0.85–1.06)
Dementia	1.0	1.2	**0.82 (0.72–0.93)**	3.2	3.3	0.98 (0.87–1.10)
Movement disorder	1.2	1.4	**0.83 (0.74–0.92)**	6.3	6.2	1.02 (0.94–1.11)
Any of the above	21.3	24.1	**0.88 (0.86–0.90)**	71..6	72.6	**0.99 (0.97–0.99)**
Negative control outcomes^a^			0.94 (0.87–1.02)			**0.92 (0.87–0.97)**

Risk ratios in bold have 95% confidence intervals not including 1.

^a^Mean of 12 negative control outcomes. Full details in Supplementary Table 6A.

### Effects of BP-CCB versus amlodipine on a subsequent neuropsychiatric diagnosis

In this analysis, we only included patients who had at least one prior psychiatric or neurodegenerative diagnosis before first exposure to a CCB. After matching, these cohorts contained 17,896 patients (Table 1 and Supplementary Table 3A). As shown in panel B of Table 2, there was a minimally lower incidence of most diagnoses during the exposure period for patients prescribed BP-CCBs compared to amlodipine (71.6% vs. 72.6%), with an overall risk ratio of 0.99 [0.97–0.99]. The only risk ratio of note for an individual disorder was for psychotic disorder (RR = 0.83 [0.74–0.94]).

### Secondary analyses

We repeated the analyses, comparing BP-CCBs with a group comprising patients prescribed either verapamil or diltiazem, both non-penetrant non-dihydropyridine CCBs (Table 3 and Supplementary Table 3B). Results were broadly similar as for the comparison with amlodipine, with the exception of dementia, for which BP-CCBs showed a similar incidence as verapamil/diltiazem in those with no prior diagnosis, and a higher incidence in those with a prior diagnosis (Table 4).

**Table 3:** A: Main baseline demographics of matched cohorts comparing BP-CCBs with verapamil or diltiazem. A: patients with no prior neuropsychiatric diagnosis. B: patients with a prior neuropsychiatric diagnosis^a^.

	A: no prior neuropsychiatric diagnosis		B: with prior neuropsychiatric diagnosis	
	BP-CCB	Verapamil or diltiazem^b^	BP-CCB	Verapamil or diltiazem^c^
Cohort size (n)	49,987	49,987	26,094	26,094
Age at index (y, SD)	59.8 (17.0)	60.4 (15.4)	57.5 (16.4)	57.7 (14.7)
Sex (M:F %)	43:57	44:56	40:60	40:60
Race (W,B,O %)^d^	56, 27, 17	54, 29, 17	59, 29, 12	58, 30, 12
Blood pressure	137/77^e^	134/76^e^	138/78^f^	133/77^f^
Body mass index (SD)	30.3 (7.0)	30.6 (7.3)	31.2 (7.6)	31.6 (8.0)
Prior psychotic disorder (%)	0	0	3	3
Prior affective disorder (%)	0	0	31	31
Prior anxiety disorder (%)	0	0	31	31
Prior substance use disorder (%)	0	0	27	26
Prior sleep disorder (%)	0	0	33	33
Prior delirium (%)	0	0	4	4
Prior dementia (%)	0	0	3	4
Prior movement disorder (%)	0	0	6	6

^a^See footnote a to Table 1.

^b^35% on verapamil, 67% on diltiazem. Numbers exceed 100% as some patients prescribed both drugs.

^c^38% on verapamil, 66% on diltiazem. Numbers exceed 100% as some patients prescribed both drugs.

^d^W: white. B: black. O: other or not known.

^e^Standard difference = 0.13.

^f^Standard difference = 0.20.

**Table 4:** Outcomes for BP-CCBs versus verapamil or diltiazem, showing percentage with each diagnosis during the exposure period, and the risk ratio. A: patients with no prior neuropsychiatric diagnosis. B: patients with a prior neuropsychiatric diagnosis.

	A: no prior neuropsychiatric diagnosis			B: with prior neuropsychiatric diagnosis		
	BP-CCB (%)	Verapamil or diltiazem (%)	Risk ratio (95% CI)	BP-CCB (%)	Verapamil or diltiazem (%)	Risk ratio (95% CI)
Psychotic disorder	0.5	0.6	**0.78 (0.66–0.93)**	2.9	3.0	0.95 (0.86–1.05)
Schizophrenia	0.2	0.2	0.90 (0.65–1.24)	1.1	1.2	0.93 (0.79–1.09)
Affective disorder	6.4	7.3	**0.88 (0.84–0.92)**	31.7	32.6	**0.97 (0.95–0.99)**
Bipolar disorder	0.4	0.5	**0.81 (0.67–0.97)**	3.6	3.7	0.97 (0.89–1.06)
Major depressive disorder	5.7	6.6	**0.87 (0.83–0.91)**	28.1	29.2	**0.97 (0.94–0.99)**
Anxiety disorder	7.1	8.6	**0.83 (0.79–0.86)**	30.2	32.5	**0.93 (0.91–0.95)**
Sleep disorder	8.9	12.0	**0.74 (0.71–0.77)**	31.6	37.2	**0.85 (0.83–0.87)**
Substance use disorder	5.2	5.4	0.95 (0.90–1.00)	23.4	23.7	0.99 (0.96–1.02)
Delirium	1.1	1.2	**0.87 (0.78–0.98)**	3.9	4.0	0.99 (0.91–1.08)
Dementia	1.1	1.0	1.03 (0.92–1.16)	3.7	3.3	**1.12 (1.03–1.23)**
Movement disorder	1.4	1.7	**0.81 (0.73–0.89)**	6.3	6.8	**0.93 (0.87–0.99)**
Any of the above	21.9	25.4	**0.86 (0.84–0.88)**	71.8	74.2	**0.97 (0.96–0.98)**
Negative control outcomes^a^			0.96 (0.89–1.02)			**0.91 (0.86–0.97)**

Risk ratios in bold have 95% confidence intervals not including 1.

^a^Mean of 12 negative control outcomes. Full details in Supplementary Table 6B.

Comparison of BP-CCBs with amlodipine divided by sex is shown in Supplementary Table 4. In people without a prior neuropsychiatric diagnosis, the profile of results was similar in men and women, though the overall risk ratio was marginally lower for women (RR = 0.85 [0.83–0.88] vs. RR = 0.92 [0.88–0.95]). In those with a prior diagnosis, there was no difference between BP-CCBs and amlodipine for men (RR = 1.01 (0.99–1.04)) and a small effect in women (RR = 0.96 (0.94–0.97)).

To explore the effect of age, we repeated the BP-CCB versus amlodipine analysis in those aged under or over 60 years old (Supplementary Table 5). Risk ratios were generally lower in the younger cohort. For those without a prior psychiatric or neurodegenerative diagnosis, the overall risk ratio was 0.82 (0.79–0.86) in the under-60s and 0.90 (0.87–0.92) in the over 60 s. In those with a prior diagnosis, the equivalent figures were 0.96 (0.94–0.98) and 0.99 (0.97–1.01), the latter reflecting no significant differences between BP-CCBs and amlodipine for any disorder in the older age group.

For the comparison between BP-CCBs and ARBs, the cohort demographics are shown in Table 5 and Supplementary Table 3C, and the results summarised in Table 6. In people with no prior neuropsychiatric diagnosis, results were variable. Some diagnoses were commoner in those prescribed BP-CCBs (e.g. psychotic disorder, RR = 1.80 [1.44–2.25] and dementia, RR = 1.27 [1.10–1.48]), whilst others were commoner with ARBs (e.g. sleep disorder, RR = 0.72 [0.69–0.75] and movement disorder, RR = 0.82 [0.73–0.93]). Overall, BP-CCBs were associated with a modestly lower risk than ARBs for any first neuropsychiatric diagnosis (RR = 0.94 [0.92–0.97]). For people with a prior neuropsychiatric diagnosis, there was no overall difference between BP-CCBs and ARBs in the incidence of any subsequent diagnosis (RR = 0.99 [0.98–1.01]), but some disorders were commoner with BP-CCBs (e.g. delirium, RR = 1.58 [1.42–1.75]) and sleep disorder was less common (RR = 0.86 [0.83–0.88]).

**Table 5:** A: Main baseline demographics of matched cohorts comparing BP-CCBs with ARBs. A: patients with no prior neuropsychiatric diagnosis. B: patients with a prior neuropsychiatric diagnosis^a^.

	A: no prior neuropsychiatric diagnosis		B: with prior neuropsychiatric diagnosis	
	BP-CCB	ARB	BP-CCB	ARB
Cohort size (n)	38,305	38,305	20,673	20,673
Age at index (y, SD)	56.8 (18.1)	57.9 (16.3)	54.9 (17.1)	55.7 (14.5)
Sex (M:F %)	42:58	43:57	41:59	43:57
Race (W,B,O %)^b^	52, 30, 18	50, 31, 19	59, 31, 10	57, 33, 10
Blood pressure	135/77	135/77	135/78	135/78
Body mass index (SD)	30.1 (7.1)	30.2 (7.3)	30.6 (7.7)	31.2 (7.7)
Prior psychotic disorder (%)	0	0	3	4
Prior affective disorder (%)	0	0	32	33
Prior anxiety disorder (%)	0	0	31	32
Prior substance use disorder (%)	0	0	31	32
Prior sleep disorder (%)	0	0	30	31
Prior delirium (%)	0	0	4	4
Prior dementia (%)	0	0	2	4
Prior movement disorder (%)	0	0	5	5

^a^See footnote a to Table 1.

^b^W: white. B: black. O: other or not known.

**Table 6:** Outcomes for BP-CCBs versus angiotensin receptor blockers (ARBs), showing percentage with each diagnosis during the exposure period, and the risk ratio. A: patients with no prior neuropsychiatric diagnosis. B: patients with a prior neuropsychiatric diagnosis.

	A: no prior neuropsychiatric diagnosis			B: with prior neuropsychiatric diagnosis		
	BP-CCB (%)	ARB (%)	Risk ratio (95% CI)	BP-CCB (%)	ARB (%)	Risk ratio (95% CI)
Psychotic disorder	0.6	0.3	**1.80 (1.44–2.25)**	3.3	2.5	**1.29 (1.16–1.45)**
Schizophrenia	0.2	0.1	**2.39 (1.54–3.72)**	1.3	1.2	1.14 (0.96–1.36)
Affective disorder	6.6	6.6	1.00 (0.95–1.06)	32.7	31.6	**1.04 (1.01–1.07)**
Bipolar disorder	0.4	0.3	1.25 (0.99–1.57)	4.1	3.5	**1.18 (1.07–1.30)**
Major depressive disorder	5.9	6.0	0.99 (0.94–1.05)	29.0	28.1	**1.03 (1.00–1.06)**
Anxiety disorder	7.3	8.0	**0.91 (0.87–0.95)**	31.1	31.0	1.00 (0.97–1.03)
Sleep disorder	8.0	11.3	**0.72 (0.69–0.75)**	28.7	33.4	**0.86 (0.83–0.88)**
Substance use disorder	5.8	4.4	**1.31 (1.23–1.39)**	28.2	24.0	**1.17 (1.14–1.21)**
Delirium	1.0	1.0	1.07 (0.93–1.23)	4.3	2.7	**1.58 (1.42–1.75)**
Dementia	1.0	0.8	**1.27 (1.10–1.48)**	3.6	2.9	**1.25 (1.12–1.39)**
Movement disorder	1.2	1.5	**0.82 (0.73–0.93)**	6.1	5.5	**1.11 (1.03–1.20)**
Any of the above	21.8	23.2	**0.94 (0.92–0.97)**	72.3	72.6	0.99 (0.98–1.01)
Negative control outcomes^a^			**0.88 (0.80–0.96)**			**0.91 (0.85–0.98)**

Risk ratios in bold have 95% confidence intervals not including 1.

^a^Mean of 12 negative control outcomes. Full details in Supplementary Table 6C.

^b^35% on verapamil, 67% on diltiazem. Numbers exceed 100% as some patients were prescribed both drugs.

^c^38% on verapamil, 66% on diltiazem. Numbers exceed 100% as some patients prescribed both drugs.

### Negative control outcomes

The negative control outcomes generally showed a lower incidence for BP-CCBs than for the comparator cohorts, with some of the differences being significant (Tables 2, 4, and 6 and Supplementary Tables 4–6). The lower incidence of most diagnoses with BP-CCB did not reflect less healthcare or opportunities for diagnosis during the exposure period, since the number of clinic visits and hospital admissions in the BP-CCB cohort was either similar to or greater than each comparator cohort (data not shown).

## Discussion

Using electronic health records we show that over a two-year period, compared to amlodipine, BP-CCBs are associated with lower risks for receiving a first diagnosis of a range of psychiatric disorders as well as for delirium, dementia and movement disorder. Results were in the same direction but much less marked for the incidence of a subsequent diagnosis in those who already had a psychiatric or neurodegenerative diagnosis before first prescription of a CCB. Effects were somewhat greater in women, and in people under 60 years old. Results were broadly similar when BP-CCBs were compared against verapamil and diltiazem. BP-CCBs also showed an overall lower incidence of first neuropsychiatric diagnoses compared to ARBs, but with several specific disorders showing greater risks.

The large size of the cohorts and the extensive matching to control for confounders argues for the robustness of the findings. However, several limitations should be noted in addition to those inherent to electronic health records research, such as errors in the accuracy or completeness of diagnostic coding, and the possibility that patients received additional healthcare outside the TriNetX network. [48, 55–57] Notably, a major concern with all pharmacoepidemiological studies is confounding by indication. For example, age, race and diabetes mellitus all affect first-line antihypertensive treatment recommendations in clinical guidelines. For the primary analysis of the present study, this should be much less of a concern since dihydropyridine CCBs have a class recommendation for use in hypertension without distinction made between individual agents. However, CCBs are also used for some other indications, and individual drugs may differ in this regard (e.g. nifedipine for Prinzmetal angina, nimodipine for subarachnoid haemorrhage). Indeed, the unmatched cohorts showed significant differences in a range of factors (data not shown), indicating that the decision to prescribe a BP-CCB rather than amlodipine is subject to a range of influences. Even after we extensively propensity-matched the cohorts the negative control outcomes still tended to show a lower incidence with BP-CCBs than with the comparator groups. This suggests some residual confounding whereby for unknown reasons (e.g. patient demand characteristics or physician behaviour), patients prescribed BP-CCBs are either generally healthier, or less likely to complain about ailments and thus get fewer diagnoses, than those prescribed the comparator agents. The fact that the number of visits and hospital admissions during the two-year period was similar or slightly higher for the BB-CCB cohort in each analysis suggests that the former explanation is more likely than the latter. Whilst the negative control outcome results do not undermine the main findings, they do emphasize the need for caution when interpreting the causality and putative mechanism of the BP-CCB advantage for brain health. Another limitation is that cohort entry was based on two prescriptions for an eligible drug separated by at least two years; however, there may not have been continuous prescriptions throughout, and neither do we know about compliance during this time. A further limitation is that, as noted earlier, brain penetrability is not an all-or-nothing property, and the nature and strength of evidence for each drug varies. Finally, the incidence of diagnoses in those with a prior neuropsychiatric disorder likely includes some patients who had a pre-existing diagnosis re-coded, rather than a true recurrence.

With these limitations in mind, an attractive interpretation of the findings is that BP-CCBs have beneficial effects on risk for psychiatric and neurodegenerative disorders by virtue of their central actions. Neuronal voltage-gated calcium channels are known to play key roles in excitation and synaptic plasticity [25, 26, 58] and have been implicated both pathophysiologically and aetiologically in various neuropsychiatric disorders [2, 10, 25, 59]. However, whether any CCBs produce significant effects on the neuronal voltage-gated calcium channels at clinically used doses has been questioned [60, 61]. Recent evidence using functional MRI provides some evidence that they do [62] but further studies are required [63]. The fact that BP-CCBs were associated with lower risk across a diverse range of disorders suggests that the mechanism involves some facet of a shared underlying brain substrate, whilst the fact that the benefits of BP-CCBs were much greater in reducing incidence of first rather than subsequent diagnoses suggests they work primarily to reduce vulnerability to the onset of illness rather than affecting their course. If this is the case, longer exposure periods than two years may be predicted to be associated with greater reductions in a first incidence of these disorders.

The average age at index in the ‘no prior diagnosis’ cohorts was 58 years (Table 1) indicating that they are a relatively resilient group and that some of the disorders (especially schizophrenia and bipolar disorder) first diagnosed in the exposure period may be atypical. However, the generalizability of the results to younger people is supported by the fact that effects were replicated – if not greater - in the sub-analysis limited to people under 60 (who had a mean age of 38 years).

The comparison of BP-CCBs with ARBs is notable, since previous studies that have considered CCBs as a single class report advantages for ARBs on many neuropsychiatric outcomes. For example, in our previous study using the same network [17], affective and anxiety disorders were both commoner with CCBs than ARBs (risk ratios 1.27 and 1.19, respectively), whereas the present data show no difference between BP-CCBs and ARBs for affective disorders, and a lower incidence of anxiety disorders. These comparisons provide complementary evidence to support relative benefits of BP-CCBs compared to other CCBs on brain health. However, BP-CCBs are associated with greater risk of psychotic disorders and dementia than ARBs, indicating that their benefits are not uniform. The basis for the differential effect of BP-CCBs and ARBs on individual disorders merits investigation, and may relate to the distribution and functions of angiotensin receptors in relevant neural circuits [64, 65].

Our findings associating BP-CCBs with a reduced incidence of neurodegenerative disorders compared to non-penetrant CCBs are complemented by similar recent epidemiological evidence for other antihypertensive drug classes. Brain-penetrant ARBs and ACEIs [65–67], and brain-penetrant β-blockers [68], are all associated with lower risks of dementia and Parkinson’s disease than their non-penetrant counterparts. These findings together encourage a broader investigation of the therapeutic potential of brain penetrability for a wide range of drugs commonly used in internal medicine.

Our data are observational and, as we have noted, significant caution is required for several reasons before drawing strong inferences. This need is highlighted by the failure of isradipine to delay progression in early Parkinson’s disease [69], despite the strong rationale from pharmacoepidemiological and preclinical findings [70, 71]. Nevertheless, the present results do suggest that BP-CCBs may be more beneficial than amlodipine, or verapamil or diltiazem, in terms of a lower risk of common psychiatric and neurodegenerative disorders. The apparent effect is not trivial, with the risk ratios indicating that BP-CCBs are associated with a 12% lower risk compared to amlodipine, and with greater differences seen for some disorders and in younger people. Appropriately designed and well-powered randomised clinical trials repurposing BP-CCBs are needed to extend recent pilot studies [72–74] and investigate this possibility with regard both to treatment and prevention. Our findings also encourage development of novel CCBs that have greater central actions and selectivity in order to enhance potency and reduce cardiovascular side effects. This is now a feasible objective, given the discovery of a repertoire of L-type voltage-gated calcium channel isoforms that are enriched in human brain [75, 76], and isoforms that are predicted to be differentially sensitive to dihydropyridine CCBs [27, 77–79].

## Supplementary information

Supplementary Table 1

Supplementary Table 2

Supplementary Table 3

Supplementary Table 4

Supplementary Table 5

Supplementary Table 6

## Data Availability

Data subject to third party restrictions.

## References

[1] Abernethy DR, Schwartz JB (1999). Calcium-antagonist drugs. N. Engl J Med.

[2] Harrison PJ, Tunbridge EM, Dolphin AC, Hall J (2020). Voltage-gated calcium channel blockers for psychiatric disorders: genomic reappraisal. Br J Psychiatry.

[3] Dubovsky SL (2019). Applications of calcium channel blockers in psychiatry: pharmacokinetic and pharmacodynamic aspects of treatment of bipolar disorder. Expert Opin Drug Metab Toxicol.

[4] Harrison PJ, Hall N, Mould A, Al-Juffali N, Tunbridge EM (2021). Cellular calcium in bipolar disorder: systematic review and meta-analysis. Mol Psychiatry.

[5] Dubovsky SL, Franks RD, Allen S, Murphy J (1986). Calcium antagonists in mania: a double-blind study of verapamil. Psychiatry Res.

[6] Hoschl C, Kozeny J (1989). Verapamil in affective disorders: a controlled, double-blind study. Biol Psychiatry.

[7] Pazzaglia PJ, Post RM, Ketter TA, Callahan AM, Marangell LB, Frye MA (1998). Nimodipine monotherapy and carbamazepine augmentation in patients with refractory recurrent affective illness. J Clin Psychopharmacol.

[8] Hollister LE, Trevino ES (1999). Calcium channel blockers in psychiatric disorders: a review of the literature. Can J Psychiatry.

[9] Cipriani A, Saunders K, Attenburrow MJ, Stefaniak J, Panchal P, Stockton S (2016). A systematic review of calcium channel antagonists in bipolar disorder and some considerations for their future development. Mol Psychiatry.

[10] Heyes S, Pratt WS, Rees E, Dahimene S, Ferron L, Owen MJ (2015). Genetic disruption of voltage-gated calcium channels in psychiatric and neurological disorders. Prog Neurobiol.

[11] Hayes JF, Lundin A, Wicks S, Lewis G, Wong ICK, Osborn DP (2019). Association of hydroxylmethyl glutaryl coenzyme A reductase inhibitors, L-Type calcium channel antagonists, and biguanides with rates of psychiatric hospitalization and self-harm in individuals with serious mental illness. JAMA Psychiatry.

[12] Boal AH, Smith DJ, McCallum L, Muir S, Touyz RM, Dominiczak AF (2016). Monotherapy with major antihypertensive drug classes and risk of hospital admissions for mood disorders. Hypertension.

[13] Cao YY, Xiang X, Song J, Tian YH, Wang MY, Wang XW (2019). Distinct effects of antihypertensives on depression in the real-world setting: A retrospective cohort study. J Affect Disord.

[14] Kessing LV, Rytgaard HC, Gerds TA, Berk M, Ekstrom CT, Andersen PK (2019). New drug candidates for depression - a nationwide population-based study. Acta Psychiatr Scand.

[15] Agustini B, Mohebbi M, Woods RL, McNeill JJ, Nelson MR, Shah RC (2020). The association of antihypertensive use and depressive symptoms in a large older population with hypertension living in Australia and the United States: a cross-sectional study. J Hum Hypertens.

[16] Kessing LV, Rytgaard HC, Ekstrom CT, Torp-Pedersen C, Berk M, Gerds TA (2020). Antihypertensive drugs and risk of depression: a nationwide population-based study. Hypertension.

[17] Colbourne L, Luciano S, Harrison PJ (2021). Onset and recurrence of psychiatric disorders associated with anti-hypertensive drug classes. Transl Psychiatry.

[18] Shaw RJ, Mackay D, Pell JP, Padmanabhan S, Bailey DS, Smith DJ (2021). The relationship between antihypertensive medications and mood disorders: analysis of linked healthcare data for 1.8 million patients. Psychol Med.

[19] Harrison PJ, Luciano S, Colbourne L (2020). Rates of delirium associated with calcium channel blockers compared to diuretics, renin-angiotensin system agents and beta-blockers: An electronic health records network study. J Psychopharmacol.

[20] Marpillat NL, Macquin-Mavier I, Tropeano A-I, Bachoud-Levi A-C, Maison P (2013). Antihypertensive classes, cognitive decline and incidence of dementia: a network meta-analysis. J Hypertens.

[21] Rouch L, Cestac P, Hanon O, Cool C, Helmer C, Bouhanick B (2015). Antihypertensive drugs, prevention of cognitive decline and dementia: a systematic review of observational studies, randomized controlled trials and meta-analyses, with discussion of potential mechanisms. CNS Drugs.

[22] Mullapudi A, Gudala K, Boya CS, Bansal D (2016). Risk of Parkinson’s disease in the users of antihypertensive agents: An evidence from the meta-analysis of observational studies. J Neurodegener Dis.

[23] Harrison PJ, Colbourne L, Luciano S (2021). Incidence of neurodegenerative and cerebrovascular diseases associated with antihypertensive drug classes. Br J Psychiatry.

[24] Ding J, Davis-Plourde KL, Sedaghat S, Tully PJ, Wang W, Philips C (2020). Antihypertensive medications and risk for incident dementia and Alzheimer’s disease: a meta-analysis of individual participant data from prospective cohort studies. Lancet Neurol.

[25] Nanou E, Catterall WA (2018). Calcium channels, synaptic plasticity, and neuropsychiatric disease. Neuron.

[26] Alves VS, Alves-Silva HS, Orts DJB, Ribeiro-Silva L, Arcisio-Miranda M, Oliveira FA (2019). Calcium signaling in neurons and glial cells: role of Cav1 channels. Neuroscience.

[27] Zamponi GW, Striessnig J, Koschak A, Dolphin AC (2015). The physiology, pathology, and pharmacology of voltage-gated calcium channels and their future therapeutic potential. Pharm Rev.

[28] Ritz B, Rhodes SL, Qian L, Schernhammer E, Olsen JH, Friis S (2010). L-type calcium channel blockers and Parkinson disease in Denmark. Ann Neurol.

[29] Lee Y-C, Lin C-H, Wu R-M, Lin J-W, Chang C-H, Lai M-S (2014). Antihypertensive agents and risk of Parkinson’s disease: a nationwide cohort study. PLoS One.

[30] Liu X, Chen C, Smith BJ (2008). Progress in brain penetration evaluation in drug discovery and development. J Pharm Exp Ther.

[31] Friden M, Winiwarter S, Jerndal G, Bengtsson O, Wan H, Bredberg U (2009). Structure-brain exposure relationships in rat and human using a novel data set of unbound drug concentrations in brain interstitial and cerebrospinal fluids. J Med Chem.

[32] Scriabine A, Schuurman T, Traber J (1989). Pharmacological basis for the use of nimodipine in central nervous system disorders. FASEB J.

[33] Bhat S, Dao DT, Terrillion CE, Arad M, Smith RJ, Soldatov NM (2012). CACNA1C (Cav1.2) in the pathophysiology of psychiatric disease. Prog Neurobiol.

[34] Siddiqi FH, Menzies FM, Lopez A, Stamatakou E, Karabiyik C, Ureshino R (2019). Felodipine induces autophagy in mouse brains with pharmacokinetics amenable to repurposing. Nat Commun.

[35] Amenta F, Sabbatini M, Strocchi P, Tomassoni D, Tayebati S, Vitali D (2001). Occupancy by oral administration of nicardipine of L-type calcium channels in rat brain. Clin Exp Hypertens.

[36] Anekonda TS, Quinn JF, Harris C, Frahler K, Wadsworth TL, Woltjer RL (2011). L-type voltage-gated calcium channel blockade with isradipine as a therapeutic strategy for Alzheimer’s disease. Neurobiol Dis.

[37] Grotta JC, Pettigrew LC, Lockwood AH, Reid C (1987). Brain extraction of a calcium channel blocker. Ann Neurol.

[38] Heffez DS, Nowak TS, Passonneau JV (1985). Nimodipine levels in gerbil brain following parenteral drug administration. J Neurosurg.

[39] Janicki PK, Siembab D, Paulo EA, Krzascik P (1988). Single-dose kinetics of nifedipine in rat plasma and brain. Pharmacology.

[40] Krol GJ, Noe AJ, Yeh SC (1984). Gas and liquid chromatography analyses of nimodipine calcium antagonist in blood plasma and cerebrospinal fluid. J Chromatogr.

[41] Larkin JG, Thompson GG, Scobie G, Forrest G, Drennan JE, Brodie MJ (1992). Dihydropyridine calcium antagonists in mice: blood and brain pharmacokinetics and efficacy against pentylenetetrazol seizures. Epilepsia.

[42] Shoemaker H, Lee HR, Roeske WR, Yamamura HI (1983). In vivo identification of calcium antagonist binding sites using [^3^H]nitrendipine. Eur J Pharm.

[43] Supavalai P, Karobath M (1984). The interaction of [3H]PY108-068 and of [3H]PN200-110 with calcium channel binding sites in rat brain. J Neural Transm.

[44] Takakura S, Sogabe K, Satoh H, Mori J, Fujiwara T, Totsuka Z (1992). Nilvadipine as a neuroprotective calcium entry blocker in a rat model of global cerebral ischemia. A comparative study with nicardipine hydrochloride. Neurosci Lett.

[45] Uchida S, Yamada S, Nagai K, Deguchi Y, Kimura R (1997). Brain pharmacokinetics and in vivo receptor binding of 1,4-dihydropyridine calcium channel antagonists. Life Sci.

[46] Urien S, Pinquier J-L, Paquette B, Chaumet-Riffaud P, Kiechel JR, Tillement JP (1987). Effect of the binding of isradipine and darodipine to different plasma proteins on their transfer through the rat blood-brain barrier. Drug binding to lipoproteins does not limit the transfer of drug. J Pharm Exp Ther.

[47] Van den Kerkhoff W, Drewes LR (1985). Transfer of the Ca-antagonists nifedipine and nimodipine across the blood-brain barrier and their regional distribution in vivo. J Cereb Blood Flow Metab.

[48] Taquet M, Dercon Q, Luciano S, Geddes JR, Husain M, Harrison PJ (2021). Incidence, co-occurrence, and evolution of long-COVID features: A 6-month retrospective cohort study of 273,618 survivors of COVID-19. PLoS Med.

[49] Austin PC (2011). An introduction to propensity score methods for reducing the effects of confounding in observational studies. Multivar Behav Res.

[50] Ali MS, Prieto-Alhambra D, Lopes LC, Ramos D, Bispo N, Ichihara MY (2019). Propensity score methods in health technology assessment: Principles, extended applications, and recent advances. Front Pharm.

[51] Haukoos JS, Lewis RJ (2015). The propensity score. JAMA.

[52] Arnold BF, Ercumen A (2016). Negative control outcomes: A tool to detect bias in randomized trials. JAMA.

[53] Lipsitch M, Tchetgen Tchetgen E, Cohen T (2010). Negative controls: a tool for detecting confounding and bias in observational studies. Epidemiology.

[54] Lin M, Aladejebi O, Hockerman GH (2011). Distinct properties of amlodipine and nicardipine block of the voltage-dependent Ca^2+^ channels Cav1.2 and Cav2.1 and the mutant channels Cav1.2/dihydropyridine insensitive and Cav2.1/dihydropyridine sensitive. Eur J Pharm.

[55] Casey JA, Schwartz BS, Stewart WF, Adler NE (2016). Using electronic health records for population health research: A review of methods and applications. Annu Rev Public Health.

[56] Cowie MR, Blomster JI, Curtis LH, Duclaux S, Ford I, Fritz F (2017). Electronic health records to facilitate clinical research. Clin Res Cardiol.

[57] Taksler GB, Dalton JE, Perzynski AT, Rothberg MB, Milinovich A, Krieger NJ (2021). Opportunities, pitfalls, and alternatives in adapting electronic health records for health services research. Med Decis Mak.

[58] Higley MJ, Sabatini BL (2012). Calcium signalling in dendritic spines. Cold Spring Harb Perspect Biol.

[59] Zamponi GW (2016). Targeting voltage-gated calcium channels in neurological and psychiatric diseases. Nat Rev Drug Disco.

[60] Spedding M, Middlemiss DM (1985). Central effects of Ca^2+^ antagonists. Trends Pharm Sci.

[61] Triggle DJ (2007). Calcium channel antagonists: clinical uses-past, present and future. Biochem Pharm.

[62] Zink CF, Giegerich M, Prettyman GE, Carta KE, van Ginkel M, O’Rourke MP (2020). Nimodipine improves cortical efficiency during working memory in healthy subjects. Transl Psychiatry.

[63] Atkinson LZ, Colbourne L, Smith A, Harmer CH, Nobre AC, Rendell J (2019). The Oxford study of Calcium channel Antagonism, Cognition, Mood instability and Sleep (OxCaMS): study protocol for a randomised controlled, experimental medicine study. Trials.

[64] Jackson L, Eldahshan W, Fagan SC, Ergul A (2020). Within the brain: the renin-angiotensin system. Int J Mol Sci.

[65] Jo Y, Kim S, Ye BS, Lee E, Yu YM (2022). Protective effect of renin-angiotensin system inhibitors on Parkinson’s disease: a nationwide cohort study. Front Pharm.

[66] Ho JK, Moriarty F, Manly JJ, Larson EB, Evans DA, Rajan KB (2021). Blood-brain barrier crossing renin-angiotensin drugs and cognition in the elderly: a meta-analysis. Hypertension.

[67] Ouk M, Wu C-Y, Rabin JS, Jackson A, Edwwards JD, Ramierez J (2021). The use of angiotensin-converting enzyme inhibitors vs. angiotensin receptor blockers and cognitive decline in Alzheimer’s disease: the importance of blood-brain barrier penetration and *APOE* ε4 carrier status. Alz Res Ther.

[68] Beaman EE, Bonde AN, Larsen SMU, Ozenne B et al. Blood-brain barrier permeable β-blockers linked to lower risk of Alzheimer’s disease in hypertension. Brain (AOL 23 February 2022). 10.1093/brain/awac076 PMC9976965 35196379

[69] Parkinson Study Group S-PDIIII. (2020). Isradipine versus placebo in early Parkinson Disease: A randomized trial. Ann Intern Med.

[70] Kang S, Cooper G, Dunne SF, Dusel B, Luan CH, Surmeier DJ (2012). Ca_V_1.3-selective L-type calcium channel antagonists as potential new therapeutics for Parkinson’s disease. Nat Commun.

[71] Liss B, Striessnig J (2019). The potential of L-Type calcium channels as a drug target for neuroprotective therapy in Parkinson’s Disease. Annu Rev Pharm Toxicol.

[72] Ostacher MJ, Iosifescu DV, Hay A, Blumenthal SR, Sklar P, Perlis RH (2014). Pilot investigation of isradipine in the treatment of bipolar depression motivated by genome-wide association. Bipolar Disord.

[73] Burdick KE, Perez-Rodriguez M, Birnbaum R, Shanahan M, Larsen E, Harper C (2020). A molecular approach to treating cognition in schizophrenia by calcium channel blockade: An open-label pilot study of the calcium-channel antagonist isradipine. Schizophr Res Cogn.

[74] Vahdani B, Kian AA, Esmaelizadeh A, Zenoozian S, Yousefi V, Mazloomzadeh S (2020). Adjunctive raloxifene and isradipine improve cognitive functioning in patients with schizophrenia: a pilot study. J Clin Psychopharmacol.

[75] Clark MB, Wrzesinski T, Garcia AB, Hall NAL, Kleinman JE, Hyde T (2020). Long-read sequencing reveals the complex splicing profile of the psychiatric risk gene CACNA1C in human brain. Mol Psychiatry.

[76] Hall NAL, Tunbridge EM (2022). Brain-enriched CACNA1C isoforms as novel, selective targets for psychiatric indications. Neuropsychopharmacology.

[77] Soldatov NM, Bouron A, Reuter H (1995). Different voltage-dependent inhibition by dihydropyridines of human Ca^2+^ channel splice variants. J Biol Chem.

[78] Liao P, Yu D, Li G, Yong TF, Soon JL, Chua YL (2007). A smooth muscle Ca_V_1.2 calcium channel splice variant underlies hyperpolarized window current and enhanced state-dependent inhibition by nifedipine. J Biol Chem.

[79] Ortner NJ, Bock G, Dougalis A, Kharitonova M, Duda J, Hess S (2017). Lower affinity of isradipine for L-type Ca^2+^ channels during substantia nigra dopamine neuron-like activity: implications for neuroprotection in Parkinson’s disease. J Neurosci.

