# Supplementary material for: Brain-penetrant calcium channel blockers are associated with a reduced incidence of neuropsychiatric disorders
Source: Mol Psychiatry. 2022 May 26;27(9):3904–12. doi: 10.1038/s41380-022-01615-6 (PMC9708561; doi:10.1038/s41380-022-01615-6)
Supplement: Supplementary file 1 — Supplementary Table 1 [file 41380_2022_1615_MOESM1_ESM.docx]

**Supplementary Table 1.** **Studies on the brain penetrance of dihydropyridine CCBs included in the present analysis.**

The list is not exhaustive, and does not include studies that only reported behavioural effects of the drugs (e.g. on memory or seizures).

| **Ref.** | **Authors** | **Drugs studied** | **Findings and conclusions** |
| --- | --- | --- | --- |
| 35 | Amenta et al 2001 | NIC | 65-70% occupancy of brain VGCCs after oral administration of NIC to rat |
| 36 | Anekonda et al 2011 | ISR | HPLC showed ISR present in mouse brain at high concentrations after chronic s.c. administration. ‘Isradipine is bioavailable to the brain…’. |
| 37 | Grotta et al 1987 | NIC | Extraction from rat brain tissue after i.c. injection. ‘…biologically active amounts of nicardipine may be available…in neurons…’ |
| 38 | Heffez et al 1985 | NIM | Binding of [^3^H]NIM after i.p. injection. ‘Accumulates quickly in the [gerbil] brain…and may be sufficient to fully saturate…even at the lowest dose administered’ |
| 39 | Janicki et al 1988 | NIF | HPLC after i.v. injection. Accumulates in rat brain, concentration dose-related and exceeds that in plasma. ‘…can easily cross the blood-brain barrier’. |
| 40 | Krol et al 1984 | NIM | Gas chromatography. CSF concentration similar to unbound plasma concentration. |
| 41 | Larkin et al 1992 | NIF, NIM | HPLC after i.p. injection in mice, and inhibition of experimental seizures. Conclude ‘NIF and NIM cross the blood brain barrier’ |
| 42 | Schoemaker et al 1983 | NIF | Displacement of [3H]nitrendipine binding in brain after i.p. injection of NIF. |
| 34 | Siddiqi et al 2019 | AML, ISR, NIC, NIF, NIM, NIS | Table 1 summarises data from PubChem and DrugBank. All drugs cross BBB except AML. |
| 43 | Supavilai and Karobath 1984 | ISR, NIF | Accumulation in brain after i.p. injection |
| 44 | Takakura et al 1992 | NIC | Brain concentration 0.3-0.8 times that in plasma after i.v. injection |
| 45 | Uchida et al 1997 | AML, ISR, NIF, NIM | Brain:heart ratio 20-fold less for AML than ISR, NIF or NIM. Specific binding in brain not seen for AML. |
| 46 | Urien et al 1987 | ISR | Extraction from brain after i.c. injection |
| 47 | Van den Kerkhoff and Drewes 1985 | NIM, NIF | Both cross BBB, NIM>NIF |

AML: amlodipine. BBB: blood brain barrier. FEL: felodipine. HPLC: high performance liquid chromatography. ISR: isradipine. NIC: nicardipine. NIF: nifedipine. NIM: nimodipine. NIS: nisoldipine.

i.c.: intra-carotid. i.p.: intra-peritoneal. i.v.: intra-venous. s.c.: sub-cutaneous.
