# Supplementary material for: Brain-penetrant calcium channel blockers are associated with a reduced incidence of neuropsychiatric disorders
Source: Mol Psychiatry. 2022 May 26;27(9):3904–12. doi: 10.1038/s41380-022-01615-6 (PMC9708561; doi:10.1038/s41380-022-01615-6)
Supplement: Supplementary file 2 — Supplementary Table 2 [file 41380_2022_1615_MOESM2_ESM.docx]

**Supplementary Table 2.** STROBE Statement.

|  | Item No. | Recommendation | Page  No. | Relevant text from manuscript |
| --- | --- | --- | --- | --- |
| **Title and abstract** | 1 | (*a*) Indicate the study’s design with a commonly used term in the title or the abstract | 2 | ...we used the TriNetX electronic health records network… |
|  |  | (*b*) Provide in the abstract an informative and balanced summary of what was done and what was found | 2 | … to identify people prescribed a brain-penetrant CCB (BP-CCB), or those given amlodipine, a CCB with low brain penetrability. We created cohorts of patients who, prior to first CCB exposure, either had to have, or could not have had, a recorded ICD-10 diagnosis in any of the following categories: psychotic disorder; affective disorder (including bipolar disorder and major depressive disorder); anxiety disorder; substance use disorder; sleep disorder; delirium; dementia, or movement disorder. Cohort pairs were propensity score matched for age, sex, race, blood pressure, body mass index, and a range of other variables. The outcomes were the incidence of these disorders measured over a two-year exposure period. Matched cohort sizes ranged from 17,896 to 49,987. In people with no prior history of psychiatric or neurodegenerative disorder, there was a significantly lower incidence of most disorders with BP-CCBs compared to amlodipine, with risk ratios ranging from 0.64 to 0.88 and an overall risk ratio of 0.88, i.e. a risk reduction of 12%. In people who did have a prior psychiatric or neurodegenerative diagnosis, differences were much smaller, but again showed lower risks for several disorders with BP-CCBs compared to amlodipine. The differences were more marked in women and in people less than 60 years old. Results were similar when comparing BP-CCBs with verapamil and diltiazem. We also compared BP-CCBs with angiotensin receptor blockers, and found an overall risk ratio of 0.94 in favour of BP-CCBs, but with differential effects across disorders including a higher risk of psychotic disorder and dementia, but a lower risk for anxiety and sleep disorders. In some analyses, there was evidence of residual confounding even after the extensive matching, in that negative control outcomes showed a reduced incidence with BP-CCBs relative to the comparator cohort. … |
| Introduction | | | |  |
| Background/rationale | 2 | Explain the scientific background and rationale for the investigation being reported | 3 | First three paragraphs of the Introduction |
| Objectives | 3 | State specific objectives, including any prespecified hypotheses | 3 | Final paragraph of the Introduction |
| Methods | | | |  |
| Study design | 4 | Present key elements of study design early in the paper | 4-6 | First four sections of Methods |
| Setting | 5 | Describe the setting, locations, and relevant dates, including periods of recruitment, exposure, follow-up, and data collection | 4-5. |  |
| Participants | 6 | (*a*) *Cohort study*—Give the eligibility criteria, and the sources and methods of selection of participants. Describe methods of follow-up  *Case-control study*—Give the eligibility criteria, and the sources and methods of case ascertainment and control selection. Give the rationale for the choice of cases and controls  *Cross-sectional study*—Give the eligibility criteria, and the sources and methods of selection of participants | 4, and ref.48 | Describes TriNetX Analytics network |
|  |  | (*b*) *Cohort study*—For matched studies, give matching criteria and number of exposed and unexposed  *Case-control study*—For matched studies, give matching criteria and the number of controls per case | 4-5, Tables 1,3,5, and Suppl. Table 3 |  |
| Variables | 7 | Clearly define all outcomes, exposures, predictors, potential confounders, and effect modifiers. Give diagnostic criteria, if applicable | 4-6 |  |
| Data sources/ measurement | 8* | For each variable of interest, give sources of data and details of methods of assessment (measurement). Describe comparability of assessment methods if there is more than one group | 4 and ref. 48 |  |
| Bias | 9 | Describe any efforts to address potential sources of bias | 4-5, 10 | Propensity score matching |
| Study size | 10 | Explain how the study size was arrived at | Tables 1,3,5 and Suppl Tables 4 and 5. | Convenience – all eligible patients in network. All sample sizes given. |

| Quantitative variables | 11 | Explain how quantitative variables were handled in the analyses. If applicable, describe which groupings were chosen and why | NA |  |
| --- | --- | --- | --- | --- |
| Statistical methods | 12 | (*a*) Describe all statistical methods, including those used to control for confounding | 5-6 |  |
|  |  | (*b*) Describe any methods used to examine subgroups and interactions | 6 |  |
|  |  | (*c*) Explain how missing data were addressed | NA |  |
|  |  | (*d*) *Cohort study*—If applicable, explain how loss to follow-up was addressed  *Case-control study*—If applicable, explain how matching of cases and controls was addressed  *Cross-sectional study*—If applicable, describe analytical methods taking account of sampling strategy | 5 | NA  Propensity score matching (p5).  NA |
|  |  | (*e*) Describe any sensitivity analyses | 6 |  |
| Results | | | | |
| Participants | 13* | (a) Report numbers of individuals at each stage of study—eg numbers potentially eligible, examined for eligibility, confirmed eligible, included in the study, completing follow-up, and analysed | 6-8 and Tables 1-6 |  |
|  |  | (b) Give reasons for non-participation at each stage | NA |  |
|  |  | (c) Consider use of a flow diagram | NA |  |
| Descriptive data | 14* | (a) Give characteristics of study participants (eg demographic, clinical, social) and information on exposures and potential confounders | 4-8, Tables 1-6 and Suppl Tables 3-5 |  |
|  |  | (b) Indicate number of participants with missing data for each variable of interest | NA |  |
|  |  | (c) *Cohort study*—Summarise follow-up time (eg, average and total amount) | 2,5,8 | Two years for everyone |
| Outcome data | 15* | *Cohort study*—Report numbers of outcome events or summary measures over time | 6-8, Tables 2,4,6 and Suppl Tables 4-6 |  |
|  |  | *Case-control study—*Report numbers in each exposure category, or summary measures of exposure |  |  |
|  |  | *Cross-sectional study—*Report numbers of outcome events or summary measures |  |  |
| Main results | 16 | (*a*) Give unadjusted estimates and, if applicable, confounder-adjusted estimates and their precision (eg, 95% confidence interval). Make clear which confounders were adjusted for and why they were included | 4-5, 6-8, Tables 2,4,6 and Suppl Tables 4-6. |  |
|  |  | (*b*) Report category boundaries when continuous variables were categorized | NA |  |
|  |  | (*c*) If relevant, consider translating estimates of relative risk into absolute risk for a meaningful time period | NA |  |

| Other analyses | 17 | Report other analyses done—eg analyses of subgroups and interactions, and sensitivity analyses | 7-8, Tables 4 and 6 and Suppl Tables 4 and 5 |  |  |  |  |
| --- | --- | --- | --- | --- | --- | --- | --- |
| Discussion | | | | | | | |
| Key results | 18 | Summarise key results with reference to study objectives | 8 | First discussion paragraph | |  |  |
| Limitations | 19 | Discuss limitations of the study, taking into account sources of potential bias or imprecision. Discuss both direction and magnitude of any potential bias | 8-9 | Second discussion paragraph | |  |  |
| Interpretation | 20 | Give a cautious overall interpretation of results considering objectives, limitations, multiplicity of analyses, results from similar studies, and other relevant evidence | 9-11 | All discussion paragraphs | |  |  |
| Generalisability | 21 | Discuss the generalisability (external validity) of the study results | 8-11 |  | |  |  |
| Other information | |  | | | | |  |
| Funding | 22 | Give the source of funding and the role of the funders for the present study and, if applicable, for the original study on which the present article is based | 11 | L.C. is supported by a Wellcome Clinical Doctoral Fellowship (grant 102176/B/13/Z). P.J.H. is supported by the National Institute for Health Research (NIHR) Oxford Health Biomedical Research Centre (BRC-2015-20005). | |  |  |
