# Supplementary material for: Brain-penetrant calcium channel blockers are associated with a reduced incidence of neuropsychiatric disorders
Source: Mol Psychiatry. 2022 May 26;27(9):3904–12. doi: 10.1038/s41380-022-01615-6 (PMC9708561; doi:10.1038/s41380-022-01615-6)
Supplement: Supplementary file 3 — Supplementary Table 3 [file 41380_2022_1615_MOESM3_ESM.docx]

**Supplementary Table 3. Full demographic details of matched cohorts**

**Supplementary Table 3A: Full baseline demographics of matched cohorts comparing BP-CCBs with amlodipine. A: patients with no prior neuropsychiatric diagnosis. B: patients with a prior neuropsychiatric diagnosis**

|  | **ICD-10 or medication code** | **A: no prior neuropsychiatric diagnosis** | |  | **B: with prior neuropsychiatric diagnosis** | |
| --- | --- | --- | --- | --- | --- | --- |
|  |  | **BP-CCB** | **Amlodipine** |  | **BP-CCB** | **Amlodipine** |
| Cohort size (n) |  | 44,731 | 44,731 |  | 17,896 | 17,896 |
| Age at index (y, SD) |  | 58.3 (17.5) | 58.8 (16.8) |  | 56.2 (17.1) | 56.0 (15.7) |
| Sex (M:F %) |  | 42:58 | 42:58 |  | 38:62 | 38:62 |
| Race (W,B,O %) |  | 50,29,21 | 50,29,21 |  | 61,26,13 | 62,26,12 |
| Blood pressure |  | 136/77 | 136/77 |  | 134/77 | 135/79 |
| BMI (SD) |  | 30.1 (6.7) | 29.8 (6.7) |  | 30.7 (7.3) | 30.4 (7.1) |
| Psychotic disorder (%) | F20-F29 | 0 | 0 |  | 3 | 3 |
| Affective disorder (%) | F30-F39 | 0 | 0 |  | 31 | 32 |
| Anxiety disorder (%) | F40-F48 | 0 | 0 |  | 30 | 31 |
| Substance use disorder (%) | F10-F19 | 0 | 0 |  | 26 | 26 |
| Sleep disorder (%) | F51, G47 | 0 | 0 |  | 32 | 32 |
| Delirium (%) | F05, R40.0, R41.0 | 0 | 0 |  | 4 | 4 |
| Dementia (%) | F01-F03, G30, G31.0, G31.2, G31.83 | 0 | 0 |  | 2 | 3 |
| Movement disorder (%) | G20-G26 | 0 | 0 |  | 6 | 6 |
| Diuretic (%) | CV700 | 15 | 15 |  | 28 | 28 |
| ACE inhibitor (%) | CV800 | 10 | 11 |  | 20 | 21 |
| ARB (%) | CV805 | 7 | 7 |  | 11 | 11 |
| β-blocker (%) | CV100 | 18 | 18 |  | 32 | 31 |
| Other antihypertensive (%) | CV490 | 9 | 6 |  | 17 | 15 |
| Antidepressant (%) | CN600 | 5 | 5 |  | 31 | 34 |
| Sedative/hypnotic (%) | CN300 | 12 | 12 |  | 37 | 39 |
| Antipsychotic (%) | CN700 | 1 | 1 |  | 7 | 8 |
| Anticonvulsant | CN400 | 4 | 4 |  | 16 | 17 |
| Gabapentin | 25480 | 3 | 3 |  | 10 | 11 |
| Pregabalin | 187832 | 1 | 1 |  | 3 | 3 |
| Lithium | CN750 | 0 | 0 |  | 0 | 1 |
| CNS stimulants | CN800 | 1 | 1 |  | 5 | 5 |
| Levodopa | 6375 | 0 | 0 |  | 1 | 1 |
| Diabetes Mellitus (%) | E08-E13 | 13 | 14 |  | 26 | 26 |
| Hypertensive disease (%) | I10-I16 | 34 | 35 |  | 65 | 65 |
| Cerebrovascular disease (%) | I60-69 | 4 | 4 |  | 11 | 11 |
| Thyroid disease (%) | E00-E07 | 6 | 6 |  | 16 | 16 |
| Ischemic heart disease (%) | I20-I25 | 7 | 7 |  | 19 | 18 |
| Disease of respiratory system (%) | J00-J99 | 16 | 16 |  | 49 | 50 |
| Disease of musculoskeletal system (%) | M00-M99 | 26 | 27 |  | 60 | 62 |
| Nicotine dependence (%) | F17 | 0 | 0 |  | 21 | 22 |
| Alcohol related disorders (%) | F10 | 0 | 0 |  | 5 | 6 |
| Problems related to socioeconomic and psychosocial circumstances (%) | Z55-Z65 | 0 | 0 |  | 3 | 3 |

**Supplementary Table 3B. A: Full baseline demographics of matched cohorts comparing BP-CCBs with verapamil or diltiazem. A: patients with no prior neuropsychiatric diagnosis. B: patients with a prior neuropsychiatric diagnosis**

|  | **ICD-10 or medication code** | **A: no prior neuropsychiatric diagnosis** | |  | **B: with prior neuropsychiatric diagnosis** | |
| --- | --- | --- | --- | --- | --- | --- |
|  |  | **BP-CCB** | **Verapamil or diltiazem** |  | **BP-CCB** | **Verapamil or diltiazem** |
| Cohort size (n) |  | 49,987 | 49,987 |  | 26,094 | 26,094 |
| Age at index (y, SD) |  | 59.8 (17.0) | 60.4 (15.4) |  | 57.5 (16.4) | 57.7 (14.7) |
| Sex (M:F %) |  | 43:57 | 44:56 |  | 40:60 | 40:60 |
| Race (W,B,O %) |  | 56, 27, 17 | 54, 29, 17 |  | 59, 29, 12 | 58, 30, 12 |
| Blood pressure |  | 137/77 | 134/76 |  | 138/78 | 133/77 |
| BMI (SD) |  | 30.3 (7.0) | 30.6 (7.3) |  | 31.2 (7.6) | 31.6 (8.0) |
| Psychotic disorder (%) | F20-F29 | 0 | 0 |  | 3 | 3 |
| Affective disorder (%) | F30-F39 | 0 | 0 |  | 31 | 31 |
| Anxiety disorder (%) | F40-F48 | 0 | 0 |  | 31 | 31 |
| Substance use disorder (%) | F10-F19 | 0 | 0 |  | 27 | 26 |
| Sleep disorder (%) | F51, G47 | 0 | 0 |  | 33 | 33 |
| Delirium (%) | F05, R40.0, R41.0 | 0 | 0 |  | 5 | 5 |
| Dementia (%) | F01-F03, G30, G31.0, G31.2, G31.83 | 0 | 0 |  | 3 | 4 |
| Movement disorder (%) | G20-G26 | 0 | 0 |  | 6 | 6 |
| Diuretic (%) | CV700 | 18 | 18 |  | 35 | 35 |
| ACE inhibitor (%) | CV800 | 12 | 12 |  | 26 | 25 |
| ARB (%) | CV805 | 9 | 9 |  | 16 | 16 |
| β-blocker (%) | CV100 | 20 | 20 |  | 38 | 37 |
| Other antihypertensive (%) | CV490 | 9 | 9 |  | 21 | 21 |
| Antidepressant (%) | CN600 | 5 | 7 |  | 31 | 33 |
| Sedative/hypnotic (%) | CN300 | 13 | 15 |  | 39 | 43 |
| Antipsychotic (%) | CN700 | 1 | 1 |  | 7 | 8 |
| Anticonvulsant | CN400 | 5 | 6 |  | 18 | 20 |
| Gabapentin | 25480 | 3 | 4 |  | 13 | 14 |
| Pregabalin | 187832 | 1 | 1 |  | 4 | 4 |
| Lithium | CN750 | 0 | 0 |  | 0 | 0 |
| CNS stimulants | CN800 | 1 | 2 |  | 5 | 6 |
| Levodopa | 6375 | 0 | 0 |  | 1 | 1 |
| Diabetes Mellitus (%) | E08-E13 | 15 | 15 |  | 30 | 30 |
| Hypertensive disease (%) | I10-I16 | 37 | 38 |  | 70 | 70 |
| Cerebrovascular disease (%) | I60-69 | 5 | 5 |  | 13 | 13 |
| Thyroid disease (%) | E00-E07 | 7 | 7 |  | 16 | 16 |
| Ischemic heart disease (%) | I20-I25 | 9 | 9 |  | 20 | 20 |
| Disease of respiratory system (%) | J00-J99 | 17 | 18 |  | 50 | 50 |
| Disease of musculoskeletal system (%) | M00-M99 | 28 | 29 |  | 62 | 62 |
| Nicotine dependence (%) | F17 | 0 | 0 |  | 21 | 21 |
| Alcohol related disorders (%) | F10 | 0 | 0 |  | 5 | 6 |
| Problems related to socioeconomic and psychosocial circumstances (%) | Z55-Z65 | 0 | 0 |  | 3 | 3 |

**Supplementary Table 3C. A: Full baseline demographics of matched cohorts comparing BP-CCBs with angiotensin receptor blockers (ARB). A: patients with no prior neuropsychiatric diagnosis. B: patients with a prior neuropsychiatric diagnosis**

|  | **ICD-10 or medication code** | **A: no prior neuropsychiatric diagnosis** | |  | **B: with prior neuropsychiatric diagnosis** | |
| --- | --- | --- | --- | --- | --- | --- |
|  |  | **BP-CCB** | **ARB** |  | **BP-CCB** | **ARB** |
| Cohort size (n) |  | 38,305 | 38,305 |  | 20,673 | 20,673 |
| Age at index (y, SD) |  | 56.8 (18.1) | 57.9 (16.3) |  | 54.9 (17.1) | 55.7 (14.5) |
| Sex (M:F %) |  | 42:58 | 43:57 |  | 41:59 | 43:57 |
| Race (W,B,O %) |  | 52, 30, 18 | 50, 31, 19 |  | 59, 31, 10 | 57, 33, 10 |
| Blood pressure |  | 135/77 | 135/77 |  | 135/78 | 135/78 |
| BMI (SD) |  | 30.1 (7.1) | 30.2 (7.3) |  | 30.6 (7.7) | 31.2 (7.7) |
| Psychotic disorder (%) | F20-F29 | 0 | 0 |  | 3 | 4 |
| Affective disorder (%) | F30-F39 | 0 | 0 |  | 32 | 33 |
| Anxiety disorder (%) | F40-F48 | 0 | 0 |  | 31 | 32 |
| Substance use disorder (%) | F10-F19 | 0 | 0 |  | 31 | 32 |
| Sleep disorder (%) | F51, G47 | 0 | 0 |  | 30 | 31 |
| Delirium (%) | F05, R40.0, R41.0 | 0 | 0 |  | 5 | 5 |
| Dementia (%) | F01-F03, G30, G31.0, G31.2, G31.83 | 0 | 0 |  | 2 | 4 |
| Movement disorder (%) | G20-G26 | 0 | 0 |  | 5 | 5 |
| Diuretic (%) | CV700 | 17 | 18 |  | 32 | 34 |
| ACE inhibitor (%) | CV800 | 15 | 17 |  | 30 | 32 |
| ARB (%) | CV805 | 0 | 0 |  | 0 | 0 |
| β-blocker (%) | CV100 | 22 | 23 |  | 39 | 40 |
| Other antihypertensive (%) | CV490 | 11 | 13 |  | 24 | 25 |
| Antidepressant (%) | CN600 | 5 | 6 |  | 32 | 35 |
| Sedative/hypnotic (%) | CN300 | 14 | 15 |  | 42 | 43 |
| Antipsychotic (%) | CN700 | 1 | 1 |  | 8 | 8 |
| Anticonvulsant | CN400 | 5 | 5 |  | 18 | 19 |
| Gabapentin | 25480 | 3 | 4 |  | 13 | 14 |
| Pregabalin | 187832 | 1 | 1 |  | 3 | 4 |
| Lithium | CN750 | 0 | 0 |  | 0 | 0 |
| CNS stimulants | CN800 | 2 | 1 |  | 5 | 6 |
| Levodopa | 6375 | 0 | 0 |  | 1 | 1 |
| Diabetes Mellitus (%) | E08-E13 | 14 | 15 |  | 28 | 28 |
| Hypertensive disease (%) | I10-I16 | 36 | 39 |  | 66 | 69 |
| Cerebrovascular disease (%) | I60-69 | 5 | 5 |  | 14 | 14 |
| Thyroid disease (%) | E00-E07 | 6 | 6 |  | 15 | 15 |
| Ischemic heart disease (%) | I20-I25 | 8 | 9 |  | 21 | 22 |
| Disease of respiratory system (%) | J00-J99 | 18 | 18 |  | 50 | 51 |
| Disease of musculoskeletal system (%) | M00-M99 | 28 | 30 |  | 61 | 63 |
| Nicotine dependence (%) | F17 | 0 | 0 |  | 24 | 26 |
| Alcohol related disorders (%) | F10 | 0 | 0 |  | 7 | 7 |
| Problems related to socioeconomic and psychosocial circumstances (%) | Z55-Z65 | 1 | 0 |  | 3 | 3 |
