# Supplementary material for: Brain-penetrant calcium channel blockers are associated with a reduced incidence of neuropsychiatric disorders
Source: Mol Psychiatry. 2022 May 26;27(9):3904–12. doi: 10.1038/s41380-022-01615-6 (PMC9708561; doi:10.1038/s41380-022-01615-6)
Supplement: Supplementary file 4 — Supplementary Table 4 [file 41380_2022_1615_MOESM4_ESM.docx]

**Supplementary Table 4. Comparison of BP-CCBs** **with amlodipine, subdivided by sex**

1. **No prior neuropsychiatric diagnosis**

|  |  | **No prior neuropsychiatric diagnosis** | | | | |
| --- | --- | --- | --- | --- | --- | --- |
|  |  | **Male** | |  | **Female** | |
|  |  | **BP-CCB** | **Amlodipine** |  | **BP-CCB** | **Amlodipine** |
| Number |  | 18,763 | 18,763 |  | 25,640 | 25,640 |
| Age at index (years) |  | 61.5 (13.9) | 62.0 (13.5) |  | 56.4 (19.2) | 56.6 (18.8) |
| Sex (M:F %) |  | 100:0 | 100:0 |  | 0:100 | 0:100 |
| Race (% W, B, O) |  | 52, 23, 25 | 51, 23, 26 |  | 48, 33, 19 | 47, 32, 21 |
| Blood pressure |  | 138/78 | 139/79 |  | 135/76 | 135/77 |
| BMI |  | 29.8 (5.8) | 30.0 (5.5) |  | 30.3 (7.1) | 29.8 (7.2) |
|  |  |  |  |  |  |  |
| **Outcomes** |  | **% in each cohort** | **Risk ratio (95% CI)** |  | **% in each cohort** | **Risk ratio (95% CI)** |
| Psychotic disorder |  | 0.4, 0.4 | 0.94 (0.69-1.28) |  | 0.4, 0.5 | 0.78 (0.60-1.01) |
| Schizophrenia |  | 0.1, 0.1 | 1.08 (0.63-1.84) |  | 0.1, 0.1 | 0.96 (0.53-1.72) |
| Affective disorder |  | 4.1, 4.9 | **0.85 (0.77-0.93)** |  | 7.8, 9.1 | **0.86 (0.81-0.91)** |
| Bipolar disorder |  | 0.3, 0.3 | 0.96 (0.66-1.41) |  | 0.5, 0.6 | **0.78 (0.61-0.98)** |
| Major depressive disorder |  | 3.7, 4.4 | **0.84 (0.76-0.93)** |  | 7.0, 8.2 | **0.86 (0.81-0.92)** |
| Anxiety disorder |  | 4.6, 5.2 | **0.89 (0.82-0.98)** |  | 8.8, 10.9 | **0.80 (0.76-0.85)** |
| Sleep disorder |  | 10.0, 10.6 | **0.94 (0.89-0.99)** |  | 7.8, 9.3 | **0.84 (0.79-0.89)** |
| Substance use disorder |  | 5.7, 6.8 | **0.84 (0.78-0.91)** |  | 4.5, 5.5 | **0.82 (0.76-0.89)** |
| Delirium |  | 1.0, 1.2 | 0.87 (0.71-1.05) |  | 0.9, 1.2 | **0.68 (0.57-0.81)** |
| Dementia |  | 0.9, 1.0 | 0.94 (0.76-1.16) |  | 1.0, 1.4 | **0.73 (0.62-0.86)** |
| Movement disorder |  | 1.2, 1.4 | 0.85 (0.71-1.01) |  | 1.2, 1.6 | **0.75 (0.64-0.86)** |
| Any of the above |  | 20.3, 22.2 | **0.92 (0.88-0.95)** |  | 22.0, 25.7 | **0.85 (0.83-0.88)** |
|  |  |  |  |  |  |  |
| Negative control outcomes |  |  | **0.88 (0.79-0.97)** |  |  | 0.94 (0.86-1.02) |

**B: With prior neuropsychiatric diagnosis**

|  | **With prior neuropsychiatric diagnosis** | | | | |
| --- | --- | --- | --- | --- | --- |
|  | **Men** | |  | **Women** | |
|  | **BP-CCB** | **Amlodipine** |  | **BP-CCB** | **Amlodipine** |
| Number | 6,795 | 6,795 |  | 10,987 | 10,987 |
| Age at index (years) | 59.4 (13.6) | 59.8 (13.1) |  | 54.6 (18.5) | 55.0 (17.1) |
| Sex (M:F %) | 100:0 | 100:0 |  | 0:100 | 0:100 |
| Race (% W, B, O) | 66, 22, 12 | 65, 23, 12 |  | 59, 29, 12 | 60, 28, 12 |
| Blood pressure | 136/78 | 138/79 |  | 133/76^a^ | 135/78^a^ |
| BMI | 30.5 (6.5) | 30.4 (6.3) |  | 30.9 (7.7) | 30.5 (7.4) |
|  |  |  |  |  |  |
| **Outcomes** | **% in each cohort** | **Risk ratio (95% CI)** |  | **% in each cohort** | **Risk ratio (95% CI)** |
| Psychotic disorder | 2.8, 3.0 | 0.93 (0.76-1.13) |  | 2.3, 2.9 | **0.80 (0.68-0.94)** |
| Schizophrenia | 1.3, 1.4 | 0.91 (0.68-1.22) |  | 0.8, 1.1 | **0.71 (0.54-0.93)** |
| Affective disorder | 25.0, 23.7 | 1.06 (0.99-1.12) |  | 37.2, 39.5 | **0.94 (0.91-0.97)** |
| Bipolar disorder | 2.8, 2.9 | 0.94 (0.78-1.15) |  | 4.2, 4.7 | 0.90 (0.80-1.02) |
| Major depressive disorder | 22.0, 20.6 | 1.07 (1.00-1.14) |  | 33.3, 35.4 | **0.94 (0.91-0.98)** |
| Anxiety disorder | 23.2, 22.4 | 1.03 (0.97-1.10) |  | 35.7, 39.0 | **0.92 (0.89-0.95)** |
| Sleep disorder | 36.8, 35.1 | 1.05 (1.00-1.10) |  | 27.8, 30.6 | **0.91 (0.87-0.95)** |
| Substance use disorder | 27.7, 27.0 | 1.03 (0.97-1.09) |  | 20.5, 21.8 | **0.94 (0.89-0.99)** |
| Delirium | 3.3, 3.7 | 0.90 (0.76-1.08) |  | 3.1, 3.6 | 0.87 (0.75-1.00) |
| Dementia | 2.9, 3.0 | 0.96 (0.79-1.17) |  | 3.4, 3.7 | 0.92 (0.80-1.06) |
| Movement disorder | 7.6, 7.1 | 1.08 (0.96-1.21) |  | 5.6, 6.3 | **0.88 (0.79-0.97)** |
| Any of the above | 71.8, 70.7 | 1.01 (0.99-1.04) |  | 71.5, 74.6 | **0.96 (0.94-0.97)** |
|  |  |  |  |  |  |
| Negative control outcomes |  | 0.95 (0.89-1.00) |  |  | 0.95 (0.88-1.02) |

^a^Standard difference for diastolic blood pressure = 0.12.
