# Supplementary material for: Brain-penetrant calcium channel blockers are associated with a reduced incidence of neuropsychiatric disorders
Source: Mol Psychiatry. 2022 May 26;27(9):3904–12. doi: 10.1038/s41380-022-01615-6 (PMC9708561; doi:10.1038/s41380-022-01615-6)
Supplement: Supplementary file 5 — Supplementary Table 5 [file 41380_2022_1615_MOESM5_ESM.docx]

**Supplementary Table 5. Comparison of BP-CCBs** **with amlodipine, subdivided by age group (18-60 years, and 61-90 years)**.

1. **No prior neuropsychiatric diagnosis**

|  |  | **No prior neuropsychiatric diagnosis** | | | | |
| --- | --- | --- | --- | --- | --- | --- |
|  |  | **Aged 18-60 years** | |  | **Aged 61-90 years** | |
|  |  | **BP-CCB** | **Amlodipine** |  | **BP-CCB** | **Amlodipine** |
| Number |  | 14,576 | 14,576 |  | 29,652 | 29,652 |
| Age at index (years) |  | 38.0 (10.7) | 37.3 (12.1) |  | 68.3 (9.4) | 68.4 (9.5) |
| Sex (M:F %) |  | 31:69 | 34:66 |  | 47:53 | 47:53 |
| Race (% W, B, O) |  | 46, 36, 18 | 46, 36, 18 |  | 51, 26, 23 | 50, 25, 25 |
| Blood pressure |  | 131/79 | 132/81 |  | 139/75 | 139/76 |
| BMI |  | 30.5 (7.4) | 30.0 (7.6) |  | 29.7 (6.1) | 29.7 (6.0) |
|  |  |  |  |  |  |  |
| **Outcomes** |  | **% in each cohort** | **Risk ratio (95% CI)** |  | **% in each cohort** | **Risk ratio (95% CI)** |
| Psychotic disorder |  | 0.3, 0.5 | **0.65 (0.45-0.93)** |  | 0.4, 0.5 | 0.83 (0.66-1.05) |
| Schizophrenia |  | 0.2, 0.2 | 0.67 (0.39-1.14) |  | 0.1, 0.1 | 0.80 (0.50-1.27) |
| Affective disorder |  | 7.4, 9.1 | **0.81 (0.75-0.88)** |  | 5.8, 6.8 | **0.85 (0.80-0.90)** |
| Bipolar disorder |  | 0.6, 1.0 | **0.65 (0.50-0.84)** |  | 0.3, 0.3 | 1.06 (0.79-1.41) |
| Major depressive disorder |  | 6.6, 7.9 | **0.84 (0.77-0.91)** |  | 5.2, 6.2 | **0.84 (0.79-0.90)** |
| Anxiety disorder |  | 9.4, 11.6 | **0.81 (0.76-0.87)** |  | 6.0, 7.2 | **0.83 (0.78-0.88)** |
| Sleep disorder |  | 7.8, 10.5 | **0.75 (0.69-0.80)** |  | 9.1, 9.8 | **0.94 (0.89-0.98)** |
| Substance use disorder |  | 6.0, 7.4 | **0.81 (0.74-0.88)** |  | 4.7, 5.5 | **0.85 (0.80-0.91)** |
| Delirium |  | 0.7, 0.8 | 0.84 (0.64-1.09) |  | 1.0, 1.3 | **0.79 (0.68-0.92)** |
| Dementia |  | 0.1, 0.1 | 0.83 (0.36-1.93) |  | 1.4, 1.8 | **0.77 (0.67-0.87)** |
| Movement disorder |  | 0.7, 1.0 | **0.72 (0.56-0.93)** |  | 1.4, 1.8 | **0.79 (0.70-0.90)** |
| Any of the above |  | 22.0, 26.8 | **0.82 (0.79-0.86)** |  | 21.1, 23.5 | **0.90 (0.87-0.92)** |
|  |  |  |  |  |  |  |
| Negative control outcomes |  |  | **0.83 (0.78-0.89)** |  |  | 0.97 (0.90-1.04) |

**B: With prior neuropsychiatric diagnosis**

|  | **With prior neuropsychiatric diagnosis** | | | | |
| --- | --- | --- | --- | --- | --- |
|  | **Age 18-60 years** | |  | **Aged 61-90 years** | |
|  | **BP-CCB** | **Amlodipine** |  | **BP-CCB** | **Amlodipine** |
| Number | 6,932 | 6,932 |  | 11,201 | 11,201 |
| Age at index (years) | 38.9 (10.2) | 38.6 (11.1) |  | 67.1 (9.3) | 67.3 (9.4) |
| Sex (M:F %) | 30:70 | 33:67 |  | 43:57 | 43:57 |
| Race (% W, B, O) | 57, 31, 12 | 58, 31, 11 |  | 64, 24, 12 | 64, 24, 12 |
| Blood pressure | 130^a^/79^b^ | 133^a^/82^b^ |  | 136/75 | 138/76 |
| BMI | 31.5 (7.8) | 31.1 (7.7) |  | 30.1 (6.8) | 30.3 (6.7) |
|  |  |  |  |  |  |
| **Outcomes** | **% in each cohort** | **Risk ratio (95% CI)** |  | **% in each cohort** | **Risk ratio (95% CI)** |
| Psychotic disorder | 2.5, 3.1 | **0.78 (0.64-0.96)** |  | 2.5, 2.8 | 0.91 (0.77-1.06) |
| Schizophrenia | 1.1, 1.3 | 0.79 (0.59-1.08) |  | 0.9, 1.1 | 0.82 (0.63-1.06) |
| Affective disorder | 36.1, 38.5 | **0.94 (0.90-0.98)** |  | 30.5, 30.4 | 1.00 (0.97-1.04) |
| Bipolar disorder | 5.6, 6.6 | **0.86 (0.75-0.98)** |  | 2.5, 2.5 | 0.99 (0.84-1.17) |
| Major depressive disorder | 32.0, 33.6 | 0.95 (0.91-1.00) |  | 27.2, 27.2 | 1.00 (0.96-1.04) |
| Anxiety disorder | 37.2, 40.0 | **0.93 (0.89-0.97)** |  | 27.2, 26.8 | 1.01 (0.97-1.06) |
| Sleep disorder | 26.9, 30.0 | **0.90 (0.85-0.95)** |  | 34.0, 33.6 | 1.01 (0.97-1.05) |
| Substance use disorder | 27.6, 30.4 | **0.91 (0.86-0.96)** |  | 20.7, 21.2 | 0.98 (0.93-1.03) |
| Delirium | 2.6, 3.2 | **0.81 (0.67-0.99)** |  | 3.7, 4.1 | 0.89 (0.78-1.01) |
| Dementia | 0.3, 0.3 | 1.06 (0.55-2.01) |  | 4.9, 5.2 | 0.95 (0.84-1.06) |
| Movement disorder | 3.3, 3.9 | 0.86 (0.72-1.02) |  | 8.2, 7.9 | 1.05 (0.96-1.14) |
| Any of the above | 71.1, 73.9 | **0.96 (0.94-0.98)** |  | 72.0, 72.7 | 0.99 (0.97-1.01) |
|  |  |  |  |  |  |
| Negative control outcomes |  | **0.90 (0.81-0.99)** |  |  | 0.95 (0.86-1.03) |

^a^Standard difference for systolic blood pressure = 0.12

bStandard difference for diastolic blood pressure = 0.15.
