# Supplementary material for: Brain-penetrant calcium channel blockers are associated with a reduced incidence of neuropsychiatric disorders
Source: Mol Psychiatry. 2022 May 26;27(9):3904–12. doi: 10.1038/s41380-022-01615-6 (PMC9708561; doi:10.1038/s41380-022-01615-6)
Supplement: Supplementary file 6 — Supplementary Table 6 [file 41380_2022_1615_MOESM6_ESM.docx]

**Supplementary Table 6. Negative control outcomes**

**Supplementary Table 6A. Negative control outcomes for BP-CCBs versus amlodipine, showing percentage with each diagnosis during the exposure period, and the risk ratio. A: patients with no prior neuropsychiatric diagnosis. B: patients with a prior neuropsychiatric diagnosis**

|  | **A: no prior neuropsychiatric diagnosis** | | |  | **B: with prior neuropsychiatric diagnosis** | | |
| --- | --- | --- | --- | --- | --- | --- | --- |
|  | **BP-CCB (%)** | **Amlodipine (%)** | **Risk ratio**  **(95% CI)** |  | **BP-CCB (%)** | **Amlodipine (%)** | **Risk ratio**  **(95% CI)** |
| Benign colonic polyp | 1.9 | 2.0 | 0.93 (0.85-1.03) |  | 2.7 | 2.9 | 0.94 (0.83-1.06) |
| Ganglion | 0.3 | 0.4 | **0.75 (0.61-0.93)** |  | 0.6 | 0.7 | 0.81 (0.63-1.05) |
| Hallux valgus | 0.5 | 0.6 | **0.83 (0.70-0.98)** |  | 0.8 | 1.0 | **0.79 (0.63-0.98)** |
| Hernia | 3.6 | 3.9 | **0.91 (0.85-0.97)** |  | 6.5 | 6.8 | 0.95 (0.88-1.03) |
| Ingrowing nail | 0.6 | 0.5 | 1.19 (0.99-1.42) |  | 1.1 | 1.0 | 1.05 (0.86-1.28) |
| Sebaceous cyst | 0.8 | 0.8 | 1.00 (0.87-1.16) |  | 1.3 | 1.4 | 0.97 (0.81-1.16) |
| Senile keratosis | 2.3 | 2.1 | **1.09 (1.00-1.19)** |  | 2.9 | 2.7 | 1.04 (0.92-1.18) |
| Trigger finger | 0.6 | 0.8 | **0.82 (0.70-0.96)** |  | 1.1 | 1.2 | 0.93 (0.76-1.12) |
| Otalgia | 1.0 | 1.1 | 0.90 (0.79-1.02) |  | 2.1 | 2.5 | **0.86 (0.75-0.98)** |
| Onycholysis | 0.3 | 0.3 | 0.96 (0.76-1.21) |  | 0.5 | 0.5 | 0.95 (0.71-1.26) |
| Viral warts | 0.7 | 0.7 | 1.04 (0.89-1.22) |  | 1.2 | 1.3 | 0.94 (0.78-1.13) |
| Cutaneous abscess | 1.1 | 1.2 | 0.91 (0.81-1.03) |  | 2.4 | 3.0 | **0.81 (0.72-0.92)** |
|  |  |  |  |  |  |  |  |
| *Average* |  |  | 0.94 (0.87-1.02) |  |  |  | **0.92 (0.87-0.97)** |

**Supplementary Table 6B. Negative control outcomes for BP-CCBs versus verapamil or diltiazem, showing percentage with each diagnosis during the exposure period, and the risk ratio. A: patients with no prior neuropsychiatric diagnosis. B: patients with a prior neuropsychiatric diagnosis**

|  | **A: no prior neuropsychiatric diagnosis** | | |  | **B: with prior neuropsychiatric diagnosis** | | |
| --- | --- | --- | --- | --- | --- | --- | --- |
|  | **BP-CCB (%)** | **Verapamil or diltiazem (%)** | **Risk ratio**  **(95% CI)** |  | **BP-CCB (%)** | **Verapamil or diltiazem (%)** | **Risk ratio**  **(95% CI)** |
| Benign colonic polyp | 2.3 | 2.5 | **0.92 (0.85-0.99)** |  | 3.1 | 3.6 | **0.85 (0.77-0.93)** |
| Ganglion | 0.3 | 0.4 | **0.78 (0.63-0.95)** |  | 0.6 | 0.7 | **0.76 (0.62-0.95)** |
| Hallux valgus | 0.6 | 0.6 | 0.97 (0.83-1.13) |  | 1.0 | 1.2 | **0.81 (0.69-0.96)** |
| Hernia | 3.9 | 4.5 | **0.85 (0.80-0.90)** |  | 6.6 | 8.1 | **0.82 (0.78-0.88)** |
| Ingrowing nail | 0.6 | 0.6 | 1.16 (0.99-1.37) |  | 1.2 | 1.2 | 1.01 (0.86-1.17) |
| Sebaceous cyst | 0.8 | 0.9 | 0.93 (0.82-1.06) |  | 1.3 | 1.3 | 0.98 (0.84-1.13) |
| Senile keratosis | 2.6 | 2.5 | 1.07 (0.99-1.16) |  | 2.8 | 2.9 | 0.99 (0.90-1.09) |
| Trigger finger | 0.7 | 0.8 | **0.84 (0.73-0.97)** |  | 1.2 | 1.3 | 0.92 (0.79-1.08) |
| Otalgia | 1.1 | 1.1 | 0.95 (0.84-1.06) |  | 2.2 | 2.3 | 0.93 (0.84-1.05) |
| Onycholysis | 0.3 | 0.4 | 0.94 (0.76-1.17) |  | 0.6 | 0.7 | 0.91 (0.73-1.12) |
| Viral warts | 0.8 | 0.7 | 1.05 (0.91-1.21) |  | 1.2 | 1.2 | 0.98 (0.83-1.14) |
| Cutaneous abscess | 1.2 | 1.1 | 1.02 (0.91-1.15) |  | 2.6 | 2.6 | 1.01 (0.91-1.12) |
|  |  |  |  |  |  |  |  |
| *Average* |  |  | 0.96 (0.89-1.02) |  |  |  | **0.91 (0.86-0.97)** |

**Supplementary Table 6C. Negative control outcomes for BP-CCBs versus angiotensin receptor blockers (ARB), showing percentage with each diagnosis during the exposure period, and the risk ratio. A: patients with no prior neuropsychiatric diagnosis. B: patients with a prior neuropsychiatric diagnosis**

|  | **A: no prior neuropsychiatric diagnosis** | | |  | **B: with prior neuropsychiatric diagnosis** | | |
| --- | --- | --- | --- | --- | --- | --- | --- |
|  | **BP-CCB (%)** | **ARB (%)** | **Risk ratio**  **(95% CI)** |  | **BP-CCB (%)** | **ARB (%)** | **Risk ratio**  **(95% CI)** |
| Benign colonic polyp | 2.0 | 2.2 | 0.94 (0.85-1.04) |  | 2.8 | 3.2 | **0.87 (0.78-0.98)** |
| Ganglion | 0.3 | 0.5 | **0.73 (0.58-0.92)** |  | 0.6 | 0.7 | 0.80 (0.63-1.02) |
| Hallux valgus | 0.5 | 0.7 | **0.75 (0.62-0.90)** |  | 0.8 | 1.1 | **0.77 (0.63-0.94)** |
| Hernia | 3.8 | 4.0 | 0.96 (0.90-1.03) |  | 6.7 | 6.7 | 0.99 (0.92-1.07) |
| Ingrowing nail | 0.6 | 0.6 | 1.11 (0.93-1.34) |  | 1.1 | 1.2 | 0.93 (0.78-1.11) |
| Sebaceous cyst | 0.7 | 0.9 | **0.81 (0.70-0.95)** |  | 1.3 | 1.5 | **0.85 (0.72-0.99)** |
| Senile keratosis | 2.2 | 2.5 | **0.89 (0.81-0.97)** |  | 2.7 | 2.8 | 0.98 (0.87-1.10) |
| Trigger finger | 0.6 | 0.9 | **0.66 (0.56-0.78)** |  | 1.0 | 1.3 | **0.76 (0.63-0.91)** |
| Otalgia | 1.1 | 1.2 | 0.91 (0.80-1.04) |  | 2.3 | 2.2 | 1.05 (0.92-1.19) |
| Onycholysis | 0.3 | 0.4 | 0.81 (0.63-1.03) |  | 0.6 | 0.6 | 0.95 (0.74-1.21) |
| Viral warts | 0.8 | 0.8 | 0.98 (0.83-1.15) |  | 1.2 | 1.2 | 0.95 (0.80-1.13) |
| Cutaneous abscess | 1.2 | 1.2 | 1.00 (0.88-1.14) |  | 2.9 | 2.7 | 1.07 (0.95-1.19) |
|  |  |  |  |  |  |  |  |
| *Average* |  |  | **0.88 (0.80-0.96)** |  |  |  | **0.91 (0.85-0.98)** |
